# Toll-Like Receptor 4: A Macrophage Cell Surface Receptor Is Activated by Trimethylamine-N-Oxide 

**DOI:** 10.22074/cellj.2021.7849

**Published:** 2021-10-30

**Authors:** Mohammad Saeed Hakhamaneshi, Alina Abdolahi, Zakaria Vahabzadeh, Mohammad Abdi, Pedram Andalibi

**Affiliations:** 1. Department of Biochemistry, Faculty of Medicine, Kurdistan University of Medical Sciences, Sanandaj, Iran; 2. Department of Molecular Medicine and Genetics, Faculty of Medicine, Kurdistan University of Medical Sciences, Sanandaj, Iran; 3. Liver and Digestive Research Center, Research Institute for Health Development, Kurdistan University of Medical Sciences, Sanandaj, Iran; 4. Cellular and Molecular Research Centre, Research Institute for Health Development, Kurdistan University of Medical Sciences, Sanandaj, Iran

**Keywords:** Macrophage, Toll-Like Receptor 4, Trimethylamine-N-Oxide

## Abstract

**Objective:**

Trimethylamine-N-Oxide (TMAO) is considered as a risk factor for atherosclerosis which further leads to inflammation during atherosclerosis. The exact mechanism(s) by which TMAO induces the inflammatory reactions remains to be determined. TMAO can cause the endoplasmic reticulum (ER) stress that triggers activation of Toll-Like Receptors (TLRs). In macrophages, this process stimulates the production of proinflammatory cytokines. This study designed to evaluate the expression level of *TLR4* in TMAO-treated macrophages.

**Materials and Methods:**

In this experimental study, different concentrations of TMAO (37.5, 75, 150, and 300 μM) were exposed to murine macrophage (J774A.1 cell line) for 8, 18, 24, and 48 hours. The cells were also treated with 2.5 mM of 4-phenyl butyric acid as well as 2µg/ml of tunicamycin respectively as negative and positive controls for inducing ER-stress. We measured the viability of treated cells by the MTT test. Besides, the expression levels of TLR4 gene and protein were evaluated using western blotting and reverse transcription- quantitative polymerase chain reaction (RT-qPCR) analysis. One-Way ANOVA was used for statistical analysis.

**Results:**

No cell death was observed in treated cells. The cells treated with 150 and 300 μM doses of TMAO for 24 hours showed a significant elevation in the protein and/or mRNA levels of *TLR4* when compared to normal control or tunicamycin-treated cells.

**Conclusion:**

Our results may in part elucidate the mechanism by which TMAO induces the macrophage inflammatory reactions in response to the induction of ER stress, similar to what happens during atherosclerosis. It also provides documentation to support the direct contribution of *TLR4* in TMAO-induced inflammation.

## Introduction

Trimethylamine N-Oxide (TMAO) is a common metabolite in humans and other species (1) mainly produced from the oxidation of Trimethylamine (TMA) by hepatic flavin-containing monooxygenase 3 (FMO3) (2). The bacterial flora of gastrointestinal tract converts choline, phosphatidylcholine, and carnitine to TMA, known as TMA/FMO3/TMAO metaorganismal pathway. Recently, TMAO has been extensively reconsidered for its role in development of several diseases including atherosclerosis and other cardiovascular diseases, non-alcoholic fatty liver (3), Alzheimer (4), type 2 diabetes mellitus (5), chronic kidney disease (6), insulin resistance (7), and gastrointestinal cancers (8). 

Nowadays, TMAO is certainly known as a new risk factor for atherosclerosis (2, 9). Atherosclerosis is a multifactorial and gradual disease that is also known as an inflammatory disorder. Macrophages are one of the main cells involved in the inflammation. The role of inflammation has been identified in all stages of diseases including onset, progression and the rupture of atherosclerotic plaques. Risk factors for atherosclerosis can lead to inflammation or exacerbation of symptoms from the beginning of life. Various risk factors for atherosclerosis including both biochemical and environmental stressor are associated with atherosclerosis pathology through a common fundamental mechanism. They all cause endoplasmic reticulum (ER) stress which in turn disrupts the proper folding of newly synthesized proteins (10). Aggregation of misfolded proteins starts the Unfolded Protein Response (UPR) pathway. UPR increases the expression of heat shock proteins (HSPs) to correct or degrade the misfolded protein (11). Prolonged stimulation of HSPs, as a danger signal, induces humoral and cellular immune responses and exacerbates inflammation of various vascular cells including macrophages (12). HSPs bind to toll like receptors (including *TLR4*) to stimulate production of inflammatory cytokines (13). TMAO has a potential to induce ER stress and activation of UPR pathway (14). It also induces the inflammatory reactions in macrophages (2, 15). Furthermore, TMA/FMO3/TMAO pathway has been documented to induce the inflammatory reactions in other cells (15-17). The detailed mechanism by which TMAO induces the inflammatory reactions in macrophages is still unclear. 

TLRs are known as cell surface receptors that is highly expressed in macrophages, T and B lymphocytes, and other cells. These molecules recognize the pathogen-associated microbial patterns (PAMPs) presented by microbial pathogens, and/or danger-associated molecular patterns (DAMPs) which have been released from dead cells. TLRs are known as a part of our innate immune system and their activation stimulates the expression of proinflammatory cytokines which consequently triggers the inflammatory reactions as well as other metabolic regulations (18-20). *TLR4* is a member of this big family that can initiate a signaling pathway resulting in production of pro-inflammatory cytokines (19). Considering the role of TMAO in activation of ER stress-induced inflammation of macrophages as well as the possible contribution of *TLR4* in this pathway, this study was designed to evaluate the amount of *TLR4* in macrophages treated with different concentrations of TMAO.

## Materials and Methods

### Cell culture

This experimental study was approved by Kurdistan University of Medical Sciences under a project number of IR.MUK.REC.1395.90. J774A.1 cell line which is a murine macrophage cell was purchased from Pasture Institute (Tehran, Iran). Cells were cultured in high glucose DMEM containing 10% fetal bovine serum, 1% penicillin-streptomycin and 4 mM L-glutamine (all from Sigma-Aldrich, USA). Cells were maintained in a cell culture incubator with sufficient humidity, at 37˚C temperature and 5% CO_2_ . Cells were treated in three separate replicates with different concentrations of TMAO including 37.5, 75, 150, and 300 µM for 8, 18, 24, and 48 hours. Another group of cells were treated with 4-phenyl butyric acid (4-PBA) at 2.5 mM concentration for 8 hours as negative control for suppressing ER stress. To provide a positive group for inducing ER stress, the same numbers of macrophages were treated with tunicamycin with a concentration of 2 µg/ml for 18 hours. Macrophages which did not receive any treatment were used as normal control group. MTT assay was applied to check the viability of the studied groups.

### MTT Test

MTT test was performed as previously described (14, 21). Briefly, in a 96-well plate, approximately 5,000 cells were seeded in each well. Different concentrations of TMAO, 4-PBA, or tunicamycin were then added to the wells in six replications for each group. Subsequently, each well was incubated with 20 ul of 3-(4, 5-Dimethylthiazol-2-yl)-2, 5-diphenyltetrazolium bromide solution for 3.5 hours. Formation of crystals was then confirmed by microscopic assessment and then each well was incubated with 100 μl of MTT solvent for 4 hours at room temperature in the dark. A microplate reader instrument (Synergy HTX, BioTek, USA) was used to measure the absorbance of each well at 570 nm. Corrected absorbance was used to calculate the viability of treated and untreated cells using the following equation: % cell viability=(mean of sample absorbance /mean of control absorbance)×100

### Western blotting

Total protein was extracted from 2.5×10^6^ cells of each group (three replicates) using cold Radio-Immunoprecipitation Assay (RIPA) lysis buffer (Sigma-Aldrich, USA). A complete protease inhibitor cocktail (at final concentration of 1 μg/ml, Santa Cruz Biotechnology, California) and PMSF (Phenyl Methyl Sulfonyl Fluoride, at final concentration of 1 mM, Sigma-Aldrich, USA) was added to lysis buffer to inhibit any protease activity. Total protein concentration was measured using bicinchoninic acid (BCA) assay. For electrophoresis, 100 μg of total protein was loaded on 12% polyacrylamide gel containing sodium dodecyl sulfate (SDS-PAGE). A semi-dry blotting for 1.5 hours at 80 mA was applied to transfer the separated proteins to a preconditioned PVDF membrane (0.2 µm, BIO-RAD, USA). To avoid a non-specific binding of antibodies, the protein-free sites of membranes were blocked by 5% skim milk. Specific primary antibodies against TLR4 (ab13867, 1:2000, Abcam, USA), and GAPDH (NB300- 328, 1: 10000, Novus Biological, UK) were exposed to membranes for 60 minutes. After three times washing with TBST solution, the membranes were incubated with the appropriate secondary antibody (HAF007, R&D, USA) for 1 hour. The chemiluminescence signals were then exposed to the X-ray film and visualized using developing and fixing solutions. The density of each band was analyzed using Image J 1.48V software. In each sample, the relative amount of TLR4 protein was normalized to the GAPDH protein. Finally, the fold change was calculated for each group relative to the control group. 

### Reverse transcription- quantitative polymerase chain reaction 

To extract total RNA, approximately 2.5×10^6^ cells were applied according to manufacturer instruction (Jena Bioscience, Germany). The quality and quantity of the extracted RNA were assessed spectrophotometrically using a Picodrop-Take3 instrument (Synergy HTX, BioTek, USA). Genomic DNA was eliminated using DNase I treatment (Scientific Inc, USA). For cDNA synthesis, a PrimeScript™ RT reagent Kit (RR037A, Takara, Japan) was applied in one cycle of three-step reactions (step 1: 15 minutes at 37˚C, step 2: 5 seconds at 85˚C, step 3: 5 minutes at 4˚C) using an Eppendorf thermal cycler (Germany). Reverse transcription-quantitative polymerase chain reaction (RT-qPCR) analysis was performed by a SYBR GREEN kit (SYBR Premix Ex Taq II kit, Tli Plus, Takara, Japan) for quantification of *TLR4* (NM_010477) and *GAPDH* (NM_008084.2) using a Corbett RG-6000 machine (Australia). For this purpose, about 50 ng of cDNA and 0.5 μl of gene-specific primers (10 pmol/µl, Table 1) were used in 40 cycles of two-step reactions (step 1: 5 seconds at 95˚C, step 2: 30 seconds at 60˚C). 

The entity of RT-qPCR assay was controlled by analysis of melting curves and gel electrophoresis of related products. LinRegPCR software (version 2013.x) was used to calculate the mRNA levels of *TLR4* and *GAPDH* (22). In each run, the relative amount of *TLR4* mRNA was normalized to *GAPDH* mRNA. 

### Statistical analysis

The relative amounts (fold change) of protein and mRNA were provided as mean ± standard error of three replication of separate measurements (n=3). SPSS software, version 20 (IBM® SPSS Inc Chicago), was used for statistical analysis. To evaluate the mean difference between the groups, One-Way ANOVA was performed. Dunnett’s test was utilized as a post-hoc test to compare the mean values of each group to the control group. P<0.05 was considered as statistically significant value.

## Results

**Fig.1:**
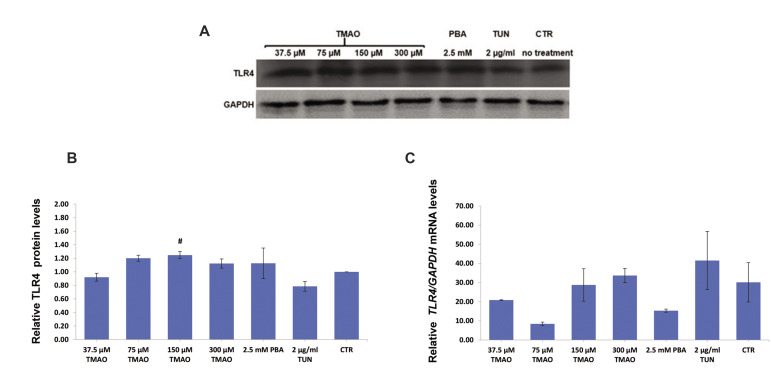
Protein and mRNA levels of TLR4 in macrophages (J774A.1 cell line) treated with TMAO and PBA (for 8 hours), and TUN (for 18 hours). **A.** Western blotting bands. **B.** Relative TLR4 protein levels. **C.** Relative *TLR4* mRNA levels. Only 150 µM of TMAO significantly increased the protein levels of TLR4. Values are mean ± standard error of three or four separate measurements. P<0.05 were considered significant. #; P<0.05 in comparison with the tunicamycin group, TMAO; Trimethylamine-N-Oxide, PBA; 4-Phenylbutyric acid, TUN; Tunicamycin, and CTR; Control.

**Fig.2:**
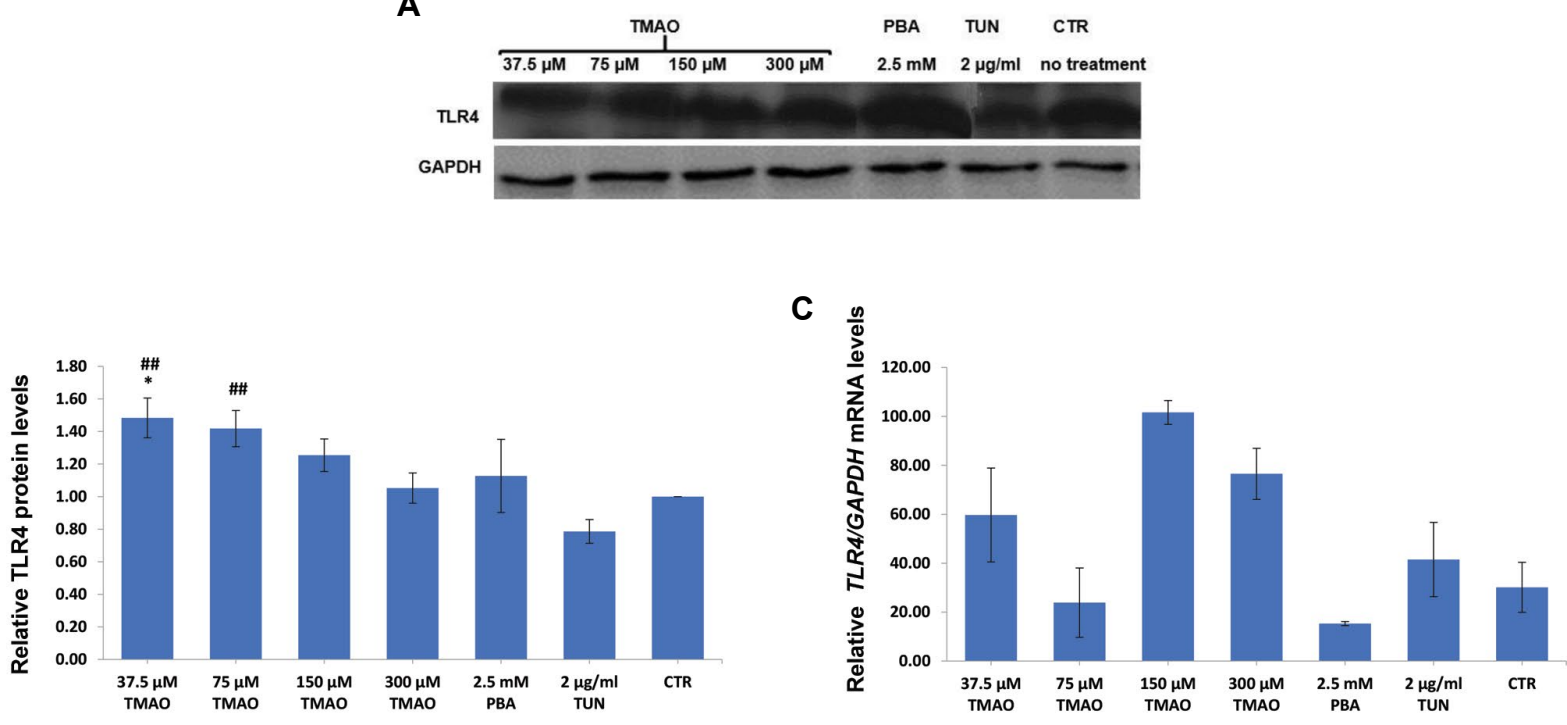
Protein and mRNA levels of TLR4 in macrophages (J774A.1 cell line) treated with TMAO and TUN (for 18 hours), and PBA (for 8 hours). **A.** Western blotting bands. **B.** Relative TLR4 levels. **C. **Relative *TLR4* mRNA levels. Only low concentrations of TMAO significantly increased the protein levels of TLR4. Values are mean ± standard error of three or four separate measurements. P<0.05 were considered significant. *; P<0.05 in comparison with control, ##; P<0.01 compared to the TUN group, TMAO; Trimethylamine-N-Oxide, PBA; 4-Phenylbutyric acid, TUN; Tunicamycin, and CTR; Control.

**Fig.3:**
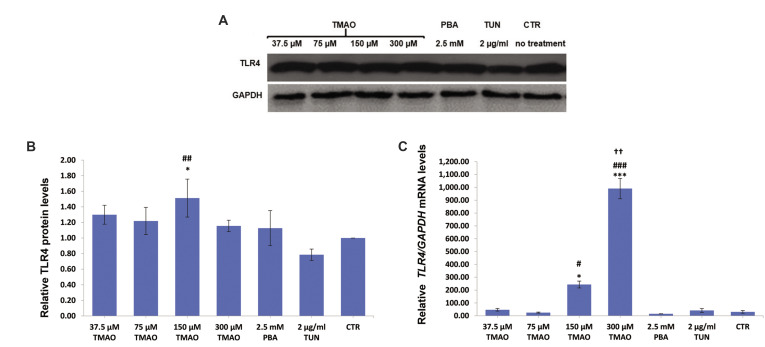
Protein and mRNA levels of TLR4 in macrophages (J774A.1 cell line) treated with TMAO (for 24 hours), PBA (for 8 hours), and TUN (for 18 hours). **A.** Western blotting bands. **B. **Relative TLR4 protein levels.** C. **Relative *TLR4* mRNA levels. Only 150 and 300 µM of TMAO significantly increased protein or mRNA levels of TLR4. Values are mean ± standard error of three or four separate measurements. P<0.05 were considered significant. *; P<0.05, ***; P<0.001 in comparison with the control, ##, ###; P<0.05, P<0.01, and P<0.001 respectively in comparison with the TUN group, ††; P<0.01 in comparison with the PBA group, TMAO; Trimethylamine-N-Oxide, PBA; 4-Phenylbutyric acid, TUN; Tunicamycin, and CTR; Control.

**Fig.4:**
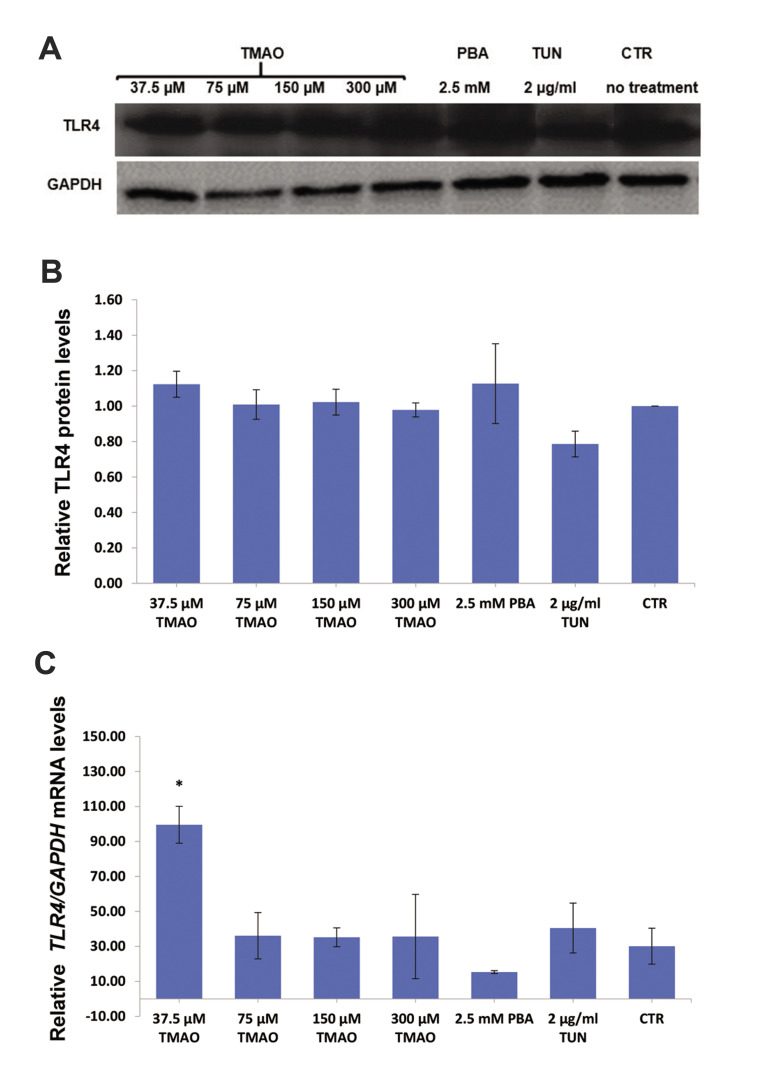
Protein and mRNA levels of TLR4 in macrophages (J774A.1 cell line) treated with TMAO (for 48 hours), PBA (for 8 hours), and TUN (for 18 hours). **A.** Western blotting bands. **B.** Relative TLR4 protein levels. **C.** Relative *TLR4* mRNA levels. Only 37.5 µM of TMAO significantly increased the mRNA levels of *TLR4*. Values are mean ± standard error of three or four separate measurements. P<0.05 were considered significant. *; P<0.05 in comparison with control, TMAO; Trimethylamine-N-Oxide, PBA; 4-Phenylbutyric acid, TUN; Tunicamycin, and CTR; Control.

To evaluate whether treatments of cells has any effects on cell viability, MTT assay was performed. The viabilities of treated and untreated cells were more than 96% suggesting no treatment-dependent cell death had occurred in macrophages. Figures 1 to 4 show the western blotting bands and the relative amounts of TLR4 at protein and mRNA levels in groups treated with TMAO, PBA and TUN for different time intervals (8, 18, 24 and 48 hours). One-Way ANOVA showed a significant difference in protein amount of TLR4 after 8 hours among TMAO-treated cells (P<0.05). Post-hoc Dunnett’s test showed that only 150 μM of TMAO significantly increased the relative amount of TLR4 protein in comparison with tunicamycin treated cells (P=0.046, Fig .1B). 

When cells were treated with TMAO for 18 hours, the relative amount of TLR4 protein was elevated. A dose of 37.5 μM TMAO significantly increased TLR4 protein amount when compared with normal control (P=0.020, Fig .2B). Besides, TMAO significantly increased the protein amount of TLR4 compared to tunicamycin treated cells at 37.5 and 75 μM concentrations (P<0.05, Fig .2B). However, *TLR4* mRNA levels were not significantly different from control group (P> 0.05, Fig .2C).

**Table 1:** Characteristics of specific primers used for reverse transcription- quantitative polymerase chain reaction (RT-qPCR)


Genes	Oligonucleotide sequences (5ˊ-3ˊ)	PCR product size (bp)	Tm (˚C)	Accession number

*TLR4*	F: ACCTGGCTGGTTTACACGTC	201	60	NM_010477
	R: CTGCCAGAGACATTGCAGAA			
*GAPDH*	F: CCATCCGGGTTCCTATAAAT	198	54	NM_008084
	R: AATCTCCACTTTGCCACTG			


After 24 hours of incubation, 150 and 300 μM of TMAO significantly increased the relative amount of *TLR4* at both protein and mRNA levels compared to CTR and/or TUN groups (P<0.05, Fig .3B, C).

TMAO-treated cells for 48 hours showed no significant difference in the relative amount of TLR4 at protein level compared to the control or tunicamycin treated cells (P>0.05, Fig .4B). Only 37.5 μM TMAO significantly increased the mRNA level of *TLR4* when compared to the control (P=0.026, Fig .4C).

These findings suggest in macrophages the expression level of *TLR4* changed in altered in a concentration and time-dependent manner when treated with TMAO (P<0.05, Figes.S1,2 , See Supplementary Online Information at celljournal.org). 

## Discussion

Macrophages play a major role in inflammation under stress conditions similar to that in atherosclerosis. In the present study, we induced ER stress in the murine macrophages (J774A.1 cell line) using TMAO as has been described previously (14). Our results showed that TMAO can induce the expression of TLR4 in macrophages, at both protein and mRNA levels, in a concentration and time-dependent manner.

The trend of *TLR4* changes in response to treatment time and concentration of TMAO was statistically significant, although it did not have a particular upward or downward direction. Cellular responses to ER stress are physiologically short term, adaptable and strongly dependent on the concentration and duration of treatment. The pattern of GRP78 changes in our previous studies also confirms this finding. GRP78 is known as the main marker of ER induction (14). The specific concentration and time for tunicamycin (2 µg/ml for 18 hours, as positive control) and 4-PBA (2.5 mM for 8 hours, as negative control) treatments used in this study, were based on our prior findings (23-26). 

In general, 24-hour treatments of TMAO led to significant elevations in expression of both TLR4 gene and protein. Where higher concentrations showed greater effects with shorter incubation times. Conversely, lower concentrations of TMAO had a greater effect when the treatment time was longer. Concentrations of 37.5, 75, and 150 μM caused a further increase in the 18- and 24- hour treatments compared to control. 

As expected, the *TLR4* mRNA pattern of changes is not fully consistent with its protein level. The stability and half-life of mRNA is shorter and more variable than protein. Therefore, for a treatment to produce a significant effect on the protein level, the mRNA level must be changed to a greater extent. However, sometimes a single mRNA molecule is used several times for translation without the need for multiple productions of mRNA transcripts. Moreover, similar to cations TMAO has a potential to form an electrostatic bond with mRNA and stabilizes its tertiary structure* in vitro*. This effect may only occurs at certain concentrations of TMAO (27). 

Our work is the first experimental (*in vitro*) study that directly measured the effect of TMAO on the expression of *TLR4* in macrophages. In this study we showed that TMAO directly induced the expression of *TLR4*. The enhancing effect of TMAO on the expression of *TLR4* has been demonstrated in cultured endothelial cells (28), cardiac fibroblast as well as in animal models (29). 

TMAO is known to correlate with the pathogenesis of different inflammatory diseases. where it plays a major role in the initiation or promotion of inflammation (3, 4, 6, 7, 8, 15-17, 30-32) . TMAO stimulates the mitogen-activated protein kinase as well as nuclear factor-κB cascade to induce the inflammatory markers (15). Furthermore, our previous work has also revealed the promoting effect of TMAO on the expression of proinflammatory cytokines in human macrophages (33).

There are several mechanisms to elucidate the role of TMAO in the development of TLR4-mediated inflammation. TLRs are the major sensors that activate cellular inflammation (34). They recognize different endogenous and exogenous ligands to stimulate the production of proinflammatory cytokine (35). TMAO is an endogenous ligand that directly interacts with TLR4 to activate the inflammation, like what happens to oxidized-low density lipoprotein (ox-LDL) (36).

TMAO is also supposed to be correlated with inflammation of macrophages through an indirect activation of TLR4 mediated by HSPs. Our previous studies have shown that TMAO induces the expression of HSPs in murine macrophages (14, 21). Extracellular form of HSPs binds to TLR4 (13). This interaction in turn stimulates the production of reactive oxygen species (ROS) and cytokines (18, 37).

TMAO-dependent production of ROS during inflammation of vascular cells was also showed by Chen et al. (38). Cluster of differentiation 36 (CD36) is another cell surface receptor of macrophages that identifies the endogenous ligands produced during atherosclerosis. It may facilitates the TLR4 signaling pathway when interacts with endogenous ligands such as TMAO (39). Wang and his colleagues showed that TMAO increases the expression of CD36 in murine macrophages (2). The ability of TMAO to alter the expression of ATP-Binding Cassette Transporter A1 (ABCA1) as well as macrophage Scavenger Receptor A1 (SRA-1) cannot be ignored for the TMAO-dependent mechanism of inflammation (40). 

## Conclusion

In macrophages TMAO treatment can induce changes in TLR4 protein and mRNA levels in a concentration and time-dependent pattern. The present study along with previous studies may in part elucidate the mechanism by which TMAO induces the macrophage inflammatory reactions in response to induction of ER stress such as what happens during atherosclerosis. From this perspective, our findings provide a documentation to support a direct contribution of TLR4 to pathogenesis of TMAO-induced macrophage inflammation. To confirm this conclusion, more detailed studies are needed to investigate the direct interaction of TMAO with *TLR4* of macrophages. Bioinformatics studies such as investigation of docking and molecular dynamics for binding TMAO to TLR4, or using specific labeled isotopes and functional assays may be useful for this purpose.

## Supplementary PDF


