# Supplementary material for: Toll-Like Receptor 4: A Macrophage Cell Surface Receptor Is Activated by Trimethylamine-N-Oxide
Source: Cell J. 2021 Oct 30;23(5):516–22. doi: 10.22074/cellj.2021.7849 (PMC8588815; doi:10.22074/cellj.2021.7849)
Supplement: Supplementary file 1 [file Cell-J-23-516-s01.pdf]

www.celljournal.org

CELL JOURNAL (Yakhteh)

Supplementary Information for

Toll-Like Receptor 4: A Macrophage Cell Surface Receptor Is Activated by Trimethylamine-N-Oxide

Mohammad Saeed Hakhamaneshi, Ph.D.[1], Alina Abdolahi, M.Sc.[2], Zakaria Vahabzadeh, Ph.D.[1, 3]*, Mohammad Abdi, Ph.D.[4], Pedram Andalibi, M.D.[1]

1. Department of Biochemistry, Faculty of Medicine, Kurdistan University of Medical Sciences, Sanandaj, Iran
2. Department of Molecular Medicine and Genetics, Faculty of Medicine, Kurdistan University of Medical Sciences, Sanandaj, Iran
3. Liver and Digestive Research Center, Research Institute for Health Development, Kurdistan University of Medical Sciences, Sanandaj, Iran
4. Cellular and Molecular Research Centre, Research Institute for Health Development, Kurdistan University of Medical Sciences, Sanandaj, Iran

Corresponding Address: P.O.Box: 6617713446, Department of Biochemistry, Faculty of Medicine, Kurdistan University of Medical Sciences, Sanandaj, Iran
Email: zakariav@yahoo.com

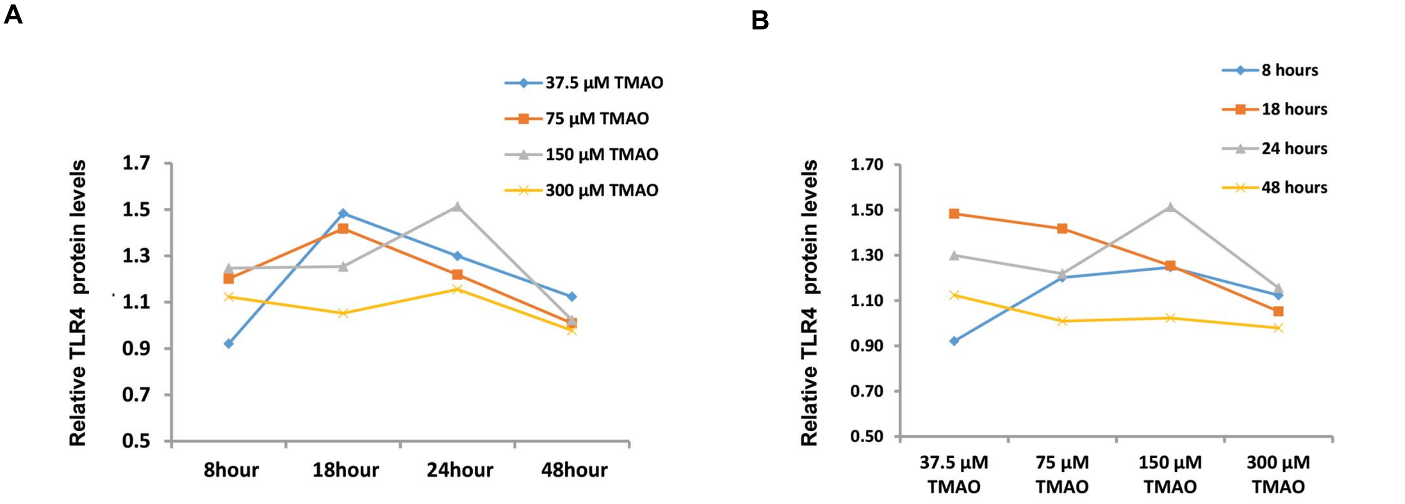

Fig.S1: Time and dose-dependent changes of TLR4 protein in TMAO treated macrophages. **A.** Time-dependent changes. **B.** Dose-dependent changes. The expression of TLR4 changed with a concentration and time-dependent pattern. TLR; Toll like receptor and TMAO; Trimethylamine-N-Oxide.

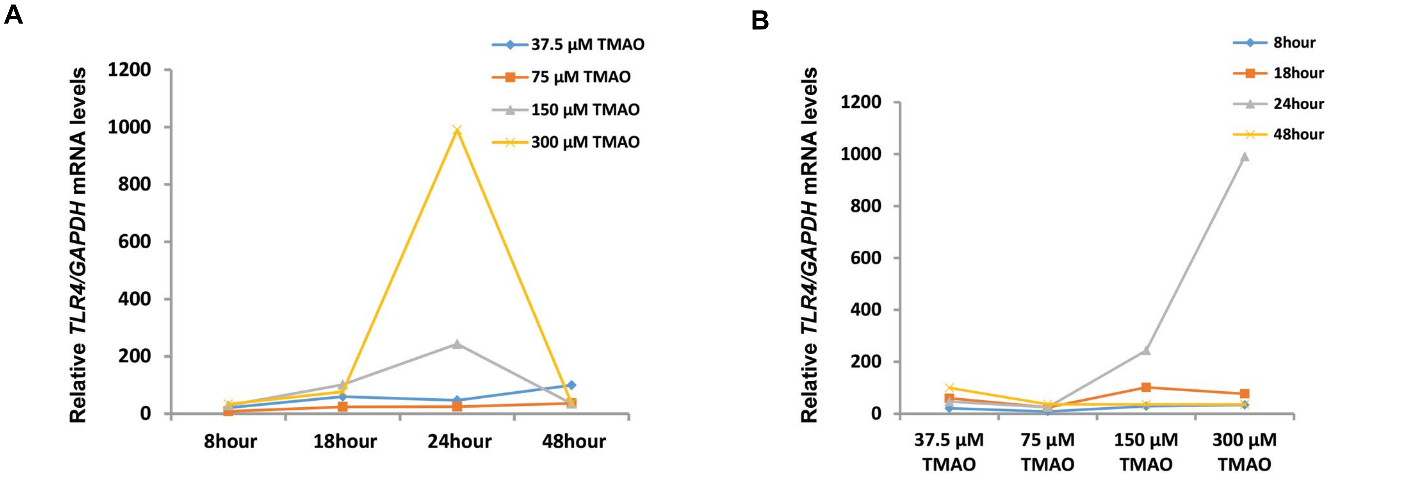

Fig.S2: Time and dose-dependent changes of *TLR4* mRNA in TMAO treated macrophages. **A.** Time-dependent changes. **B.** Dose-dependent changes. The expression of *TLR4* changed with a concentration and time-dependent pattern. TLR; Toll like receptor and TMAO; Trimethylamine-N-Oxide.
